# Simple and precise targeted editing of the human genome using rAAV-mediated homologous recombination

**DOI:** 10.1186/1753-6561-6-S6-P51

**Published:** 2012-10-01

**Authors:** Christopher J Torrance

**Affiliations:** 1 Horizon Discovery Ltd, Cambridge Research Park, Cambridge, CB25 9TL, UK

## 

The rate limiting step in translating normal and pathological human genetic variations into a deeper understanding of which are functionally relevant, and the development of genetically defined disease models that can speed up and rationalize the discovery of novel targeted therapies, now lies firmly with our ability to quickly and precisely alter any sequence in the human genome, just as we have been able to do in mice and other lower organisms for many years. However, routinely altering any user-defined endogenous DNA sequence in somatic human cells has been historically challenging due to their uniformly low levels of homologous recombination.

Horizon's GENESIS™ gene-editing platform [http://www.horizondiscovery.com] uses the unique ability of targeted recombinant adeno-associated viral (rAAV) vectors to naturally stimulate and piggyback on the high-fidelity homologous recombination DNA-repair pathway in human cells, without the requirement for eliciting double-stranded DNA breaks using exogenous nucleases, to precisely engineer any DNA variation of choice into any human cell line, including the introduction of subtle point mutations and single nucleotide polymorphisms (SNPs), just as they occur in real patients.

The ability to rapidly, accurately and stably engineer human genomes without introducing confounding off-target effects has the potential to transform the field of functional genomics and the design of future targeted, or personalized, therapies. Data will be presented highlighting the use of GENESIS to create genetically defined 'X-MAN' (gene X, mutant and normal) human isogenic cell-line pairs, which accurately model specific genetic variations present in a defined cancer patient population, and provide a matched normal genetic background for reference, in key application areas. First, defining the core phenotype driven by specific cancer genes; second, profiling the activity and therapeutic window of novel cancer therapies on specific target patient genotypes; third, systematically de-orphaning the cancer genome with next-generation'synthetically lethal'drug targets by combining with RNA interference and chemical genomics tools; and fourth, their use as gold-standard genomic reference materials for benchmarking molecular diagnostic assays and platforms.

